# Mr. Turnbull, on the Cure of Hydrophobia

**Published:** 1808-02

**Authors:** W. Turnbull

**Affiliations:** Warwick-Court, Holborn


### *To the Editors of the Medical and Physical Journal.*

GENTLEMEN,

AS there have been several recent instances of persons having been bitten by mad dogs, some of whom have fallen victims to that most dreadful of all maladies, the HYDROPHOBIA, — the following attested case, successfully treated by the late Dr. Turnbull, and which was published by him in the Gazetteer of 1784, may not at this time be unacceptable to your Readers. I have sent an abridged account of it, from the manuscript, for insertion, if you should consider it deserving a place in your useful and extensive publication.

I am, &c.

*Warwick-Court, Holborn,*

*Dec.* 23, 1807.

W. TURNBULL.

---

CASE OF HYDROPHOBIA.

Robert Dixon, a weaver, of Norham Mains, Northumberland, was bitten by a mad dog on the 30th of July, 1761, and what is very remarkable in this case, was attacked *on the same day* he received the injury, with pains in the wound which the rabid animal had made on the outside of his leg. These pains extended to the knee, and gradually upwards to his thigh, from thence to his stomach, occasioning sickness and great weight and oppression about his chest. These sensations daily increased to the 18th of August, when every symptom became more severe, succeeded with most violent convulsions and spasms, which threatened immediate suffocation, particularly when water or any other fluid was presented to him. These symptoms were accompanied with every other dreadful indication of *Hydrophobia.* That Robert Dixon was perfectly recovered in the September following; and at the time this account was first published, was living, and in good health, being a period of 23 years after the injury was received. The mode of treatment observed in this case was as follows: 1st. To the wounded extremity was applied a caustic, which was kept open by blistering and other stimulating ointments, from the first appearance of the disease, till some time after the cure. 2nd. The leg was frequently bathed with warm oil. 3d. A tea-spoonful of the following electuary was administered three times a-day, composed of Peruvian bark, two ounces; valerian in powders one ounce; cinabar of antimony, one ounce; musk, half a drachm; camphor, one drachm; conserve of wormwood, four ounces; syrup of saffron, a sufficient quantity to form an electuary. Opium was also liberally administered to allay the existing irritations and spasms. To the throat was applied a plaster composed of opium, camphor, musk, assafœtida, and gum galbanum. The last application seemed to be more effectual than any other medicine administered. On recovering his speech, he declared he found the greatest relief from this plaster, and that in a short time after it was applied, he experienced an agreeable and soothing warmth, which descended from the throat to his leg, in the same course and direction in which the spasmodic strictures ascended, and all the complicated and painful symptoms progressively diminished, until a perfect recovery was accomplished. It is necessary here to mention, that as a farther proof of the efficacy of this medicine, it was not had recourse to until the symptoms of Hydrophobia had evidently taken place, which did not occur until the 18th of August, as before observed. As this external application to the throat was attended with such happy success in the most dreadful stage of the disease, might it not have prevented this most aweful visitation altogether, had it been applied so soon as possible after the bite was received? It would certainly be recommendable at least, to give it a trial; particularly when the mouth, throat, and other parts engaged in deglutition, are principally of this disease; and as this malady, in my opinion, bears a strong affinity to nervous and spasmodic diseases, the remedies above detailed require our most serious consideration, especially when they have been attended with such success.

Does not the croup, a disease which first appeared about 60 years in the North-East coast of Northumberland, and which, for the most part, proves fatal, and the globus hystericus in women, bear some analogy to the symptoms of Hydrophobia?

N. B. It is a fact not to be contradicted, that there are only two cases authenticated and recorded in the Annals of British Medicine, where persons have been seized with Hydrophobia, and afterwards recovered, viz. in the present instance of Robert Dixon, and in the case of Elizabeth Bryant, as published by the late Dr. Nugent of Bath.

